# Correction: Unveiling concentration effects on the structural and optoelectronic characteristics of Zn_1−*x*_Cd_*x*_S (*x* = 0, 0.25, 0.50, 0.75, 1) cubic semiconductors: a theoretical study

**DOI:** 10.1039/d2ra90084j

**Published:** 2022-09-16

**Authors:** Muhammad Aamir Iqbal, Maria Malik, Abu Zahid, Md. Rasidul Islam, Iván D. Arellano-Ramírez, Mohammed Al-Bahrani

**Affiliations:** Centre of Excellence in Solid State Physics, University of the Punjab Lahore 54590 Pakistan aamir.hum@gmail.com; School of Materials Science and Engineering, Zhejiang University Hangzhou 310027 China; Department of Computer Science, Lamar University Beaumont Texas-77705 USA; Department of Electrical and Electronic Engineering, Bangamata Sheikh Fojilatunnesa Mujib Science and Technology University Melandah Jamalpur-2012 Bangladesh; Department of Physics, Universidad Tecnológica de Pereira Pereira 660003 Colombia USA; Air Conditioning and Refrigeration Techniques Engineering Department, Al-Mustaqbal University College Babylon 51001 Iraq

## Abstract

Correction for ‘Unveiling concentration effects on the structural and optoelectronic characteristics of Zn_1−*x*_Cd_*x*_S (*x* = 0, 0.25, 0.50, 0.75, 1) cubic semiconductors: a theoretical study’ by Muhammad Aamir Iqbal *et al.*, *RSC Adv.*, 2022, **12**, 22783–22791, https://doi.org/10.1039/D2RA03850A.

The authors regret that incorrect details were given for ref. 4 in the original article. The correct version of ref. 4 is given below.

M. A. Iqbal, M. Malik, W. Shahid, S. Irfan, A. C. Alguno, K. Morsy, R. Y. Capangpangan, P. V. Pham and J. R. Choi, Ab-initio study of pressure influenced elastic, mechanical and optoelectronic properties of Cd_0.25_Zn_0.75_Se alloy for space photovoltaics. *Sci. Rep.*, 2022, **12**, 12978.

The Royal Society of Chemistry apologises for these errors and any consequent inconvenience to authors and readers.

## Supplementary Material

